# A Reevaluation of the Native American MtDNA Genome Diversity and Its Bearing on the Models of Early Colonization of Beringia

**DOI:** 10.1371/journal.pone.0003157

**Published:** 2008-09-17

**Authors:** Nelson J. R. Fagundes, Ricardo Kanitz, Sandro L. Bonatto

**Affiliations:** 1 Faculdade de Biociências, Pontifícia Universidade Católica do Rio Grande do Sul, Porto Alegre, Rio Grande do Sul, Brazil; 2 Departamento de Genética, Instituto de Biociências, Universidade Federal do Rio Grande do Sul, Porto Alegre, Rio Grande do Sul, Brazil; University of Utah, United States of America

## Abstract

The Americas were the last continents to be populated by humans, and their colonization represents a very interesting chapter in our species' evolution in which important issues are still contentious or largely unknown. One difficult topic concerns the details of the early peopling of Beringia, such as for how long it was colonized before people moved into the Americas and the demography of this occupation. A recent work using mitochondrial genome (mtDNA) data presented evidence for a so called “three-stage model” consisting of a very early expansion into Beringia followed by ∼20,000 years of population stability before the final entry into the Americas. However, these results are in disagreement with other recent studies using similar data and methods. Here, we reanalyze their data to check the robustness of this model and test the ability of Native American mtDNA to discriminate details of the early colonization of Beringia. We apply the Bayesian Skyline Plot approach to recover the past demographic dynamic underpinning these events using different mtDNA data sets. Our results refute the specific details of the “three-stage model”, since the early stage of expansion into Beringia followed by a long period of stasis could not be reproduced in any mtDNA data set cleaned from non-Native American haplotypes. Nevertheless, they are consistent with a moderate population bottleneck in Beringia associated with the Last Glacial Maximum followed by a strong population growth around 18,000 years ago as suggested by other recent studies. We suggest that this bottleneck erased the signals of ancient demographic history from recent Native American mtDNA pool, and conclude that the proposed early expansion and occupation of Beringia is an artifact caused by the misincorporation of non-Native American haplotypes.

## Introduction

The Americas were the last continents to be settled by modern humans, most probably from northeast Asia through Beringia, the landmass that connected Asia and the Americas during periods of low sea-level [1]. Archeological data suggest that the continent was colonized in the late Pleistocene after the Last Glacial maximum (LGM). The oldest sites for North and South America are about 14.5 ky old [1], possibly suggesting a fast southward movement of the initial settlers. However, the scarceness of late Pleistocene human remains in northeast Asia make it difficult to evaluate the details of the population processes in Beringia that ultimately led to the peopling of the New World.

As an alternative to the study of archeological data, molecular data have been extensively used to infer when and how modern humans colonized the world reviewed in [2], [3]. Since the pioneering study by Cann et al. [4], the mitochondrial DNA (mtDNA) has become the most widely used genetic marker to study recent human evolution [5]. In Native Americans, early studies of mtDNA variation found that these populations have five distinct major mtDNA haplogroups (A, B, C, D and X) [6], [7], all of Asian origin. Although most of these studies seemed to converge on a model suggesting a single pre-Clovis migration [8]–[10], no consensus emerged for details such as the timing and pace of the putative occupation event under this scenario. These controversies notwithstanding, the divergence between Native American and Asian sequences for each mtDNA haplogroup led some authors to suggest that the Native American founder population stayed isolated in Beringia from the remaining Asian populations prior to their entry in the Americas [9], the so called “out of Beringia” model. Under this scenario, Beringia played a key-role in the differentiation of the mtDNA haplogroups presently found in Native Americans.

The study of complete mtDNA sequences from Native Americans [11]–[14] has allowed investigators to examine mtDNA variation in the New World with much greater resolution. The first systematic survey of coding-region mtDNA sequences including individuals from Native American ancestry was carried by Herrnstadt et al. [11], who studied individuals sampled from an urban population and relied on the screening of an incomplete set of HVS-I markers to identify mtDNA haplotypes as being of Native American origin [15]. Afterwards, a thorough revision of these sequences partially changed the original classification of the Native American genomes [15]. Further studies, using samples obtained mainly from native American populations, revealed new putative founder haplotypes [12], [14], and suggested an average coalescence time for the most common haplogroups of around 13.5 thousand years ago (kya) or 19 kya depending on the estimates being based on only synonymous transitions [16] or on all substitutions [17], respectively. The most extensive work to date [13] showed that all five major haplogroups have a similar pattern of genetic diversity, and that they expanded together towards the end of the last glacial maximum (LGM) around 18 kya.

The development of new analytical methods allowed the estimation of the past demography changes using the Bayesian Skyline Plot (BSP) approach, which allows inference of past population size changes without assuming any a priori demographic scenario [18]. Thus, so far, four roughly synchronous studies [13], [19]–[21], using partially overlapping data sets, applied such an analysis using putative Native American mtDNA genomes. Adjusting for the different substitution rates used, all of them showed evidence for quick and strong population growth in the late Pleistocene, likely near the end of the LGM, preceded by a population bottleneck that lasted for a few thousand years e.g., [13].

Interestingly, only one of these studies concluded that the BSP of Native American mtDNAs suggested an additional and more ancient period of population growth followed by a long period of population stability [20]. The authors interpreted these results as representing the expansion out of Central Asia into Beringia after divergence from Asians (∼43–36 kya) followed by a long period (∼20,000 years) of population stability in Beringia and finally by the strong population growth stage (∼16 kya) after the LGM associated to the peopling of the Americas. They called this scenario the “three-stage” colonization model for the peopling of the Americas, even though several of the more recent colonization models could likewise be described as having “three stages”: a first stage from Asia into Beringia, a second stage of isolation in Beringia and a third stage with an expansion out of Beringia into the Americas [9], [10], [12]–[14], [15]. These studies highlight the importance of a stage in Beringia prior to the peopling of the Americas, and one of them even provided a rough estimate of the time the Native American founder population spent in Beringia using the number of diagnostic substitutions found in Native American mtDNA sub-haplogroups [13]. However, what differentiates the study of Kitchen et al. [20] from the others is that it was the only one that estimated a detailed demographic history for the first two stages, although it used evidence and methods (BSP) similar to those employed by the other works. Nonetheless, one possible problem with this study is that the data set of mtDNA genomes they used consisted primarily of the original data set of Herrnstadt et al. [11], in which several mtDNA genomes regarded as Native American are most likely of non-Native ancestry [15] and which also includes several sequence errors detected recently but not corrected in the mtDB database that they used [22], [23].

In this study, we provide a reanalysis of the BSP results from Kitchen et al. [20] using a rigorous criterion for defining Native American ancestry [12], [14], [15] to determine if the specific three-stage model suggested by these authors is still supported when a more reliable data set is used. We also investigate the likely source of the early expansion detected by that study.

## Materials and Methods

### Data sets

Initially, the original Kitchen et al. [20] data set (n = 77) was used to replicate their original findings using the evolutionary model specified in that report and another one (see below). Based on information detailed in Bandelt et al. [15] and Achilli et al. [14], we removed seven individuals who are very likely of Asian ancestry. These include three individuals assigned to haplogroup E in Herrnstadt et al. (Figure 2 in [11]), as well as four individuals belonging to the Asian sub-haplogroups B1 or B4c [15]. Another individual (Herrn552; see [11]) was removed, since it actually belongs to the West European haplogroup H. This individual was probably incorporated into the data set by mistake, as individual 532 (from Native American haplogroup A2) is absent from their data set. Finally, another individual (Kiv2870) was removed, since it had all the diagnostic coding-region mutations for West European sub-haplogroup X2b (8393, 13708, 15927), and none of the diagnostic coding-region mutations for Native American sub-haplogroup X2a (8913, 12397, 14502), and thus it is likely of recent European ancestry. That modifications resulted in a new, corrected, data set of 68 sequences of likely Native American origin, and, in a third data set with only the 9 sequences which have been misincorporated in the original data set of 77 sequences analyzed by Kitchen et al. [20]. Finally, to better test whether the anomalous early expansion seen in Kitchen et al. [20] BSP results could be explained by the non-Native American mtDNA genomes, we created a set of 10 alignments with nine genomes each randomly selected from the mtDNA genomes from the macrohaplogroups M and N [24].

### Bayesian Skyline Plot

BSPs [18] have been constructed in the program Beast 1.4.7 (http://beast.bio.ed.ac.uk/). For all analyses, Markov Chain Monte Carlo (MCMC) samples were based on 100,000,000 generations, logging every 2,500 steps, with the first 10,000,000 generations discarded as the burn-in. All analyses were run multiple times to check for convergence. Following Fagundes et al. [13], we used the HKY+Γ evolutionary model, a log-normal relaxed molecular clock with a mean substitution rate of 1.26×10^−8^ mutations/site/year [17] for the complete coding sequence. The scaled effective population size was converted to the effective female population size N_ef_, assuming a generation time of 25 years. Importantly, assumptions about the mutation rate and the generation time will only affect the scale of the BSP, but not its shape.

## Results

**Figure 1:**
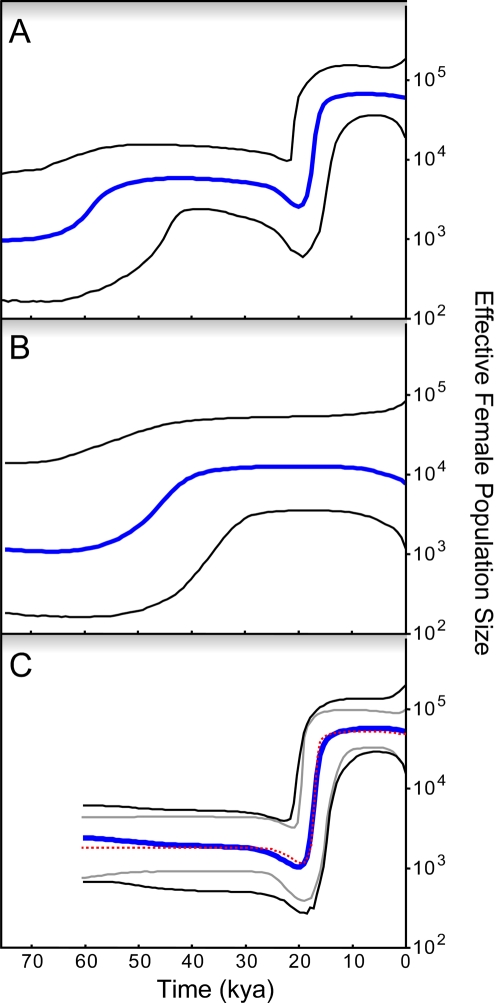
Bayesian Skyline Plots using different sequence sets. BSPs estimated with 100 million MCMC iterations sampled every 2,500 steps using log-normal relaxed clock and HKY model plus gamma (eight categories) with the standard substitution rate of 1.26×10^−8^ sites/yr and a generation time of 25 yr. The *y* axis represents the female effective population size in a log scale and the *x* axis shows time in thousands of years ago (kya). The thicker blue lines are the median for population size and the thinner black lines represent the 95% higher posterior density (HPD) intervals. (A) BSP using the original 77 individuals from [20]. (B) BSP for the nine misincorporated non-Native American sequences. (C) BSP for the 68 confirmed Native American haplotypes in [20] in blue and black; and the BSP from [13] in red dashed (median) and gray lines (95% HPD interval).

Our analysis of the BSP for the original Kitchen et al. data set [20] reproduced their original results, which was two periods of population growth (Figure 1A) with a long stasis between them. However, the corrected data set provided evidence for a single, post-LGM, population growth (Figure 1C), in close accordance to the other BSP analyses using only mtDNA of Native American origin [13], [19], [21]. That is, there was only a long tail of roughly constant population size between the time for most recent common ancestor (TMRCA) of each Native American haplogroup around the LGM and the sample TMRCA.

**Figure 2:**
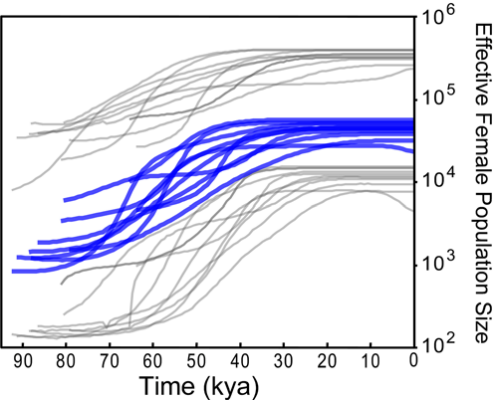
Bayesian Skyline Plot for ten replicates of nine random non-African haplotypes. Ten BSPs using random samples of nine non-African individuals from [24] belonging to macrohaplogroups M and N, showing a similar pattern of expansion between ∼80 and ∼40 kya. All BSPs were calculated with 100 million MCMC generations sampling every 2,500 using the same model applied to the BSPs in Figure 1. Axes and lines are as in Figure 1.

More strikingly, when we considered only those individuals which were removed from the original data set, the signal for the earlier expansion reappeared, despite the very low sample size in this data set (n = 9) (Figure 1B). Using our substitution rate, this expansion began ∼60 kya. These results clearly show that the signal for a population expansion that they detected [20] in Native Americans mtDNAs much before LGM is an artifact caused by the incorporation of non-Native American haplotypes into the analysis. Since these nine haplotypes seem to be of Asian and European origin, from macrohaplogroups M and N, we conjecture that this signal of expansion may be related to the demographic expansion out-of-Africa that gave rise to Eurasians. The BSPs of the ten data sets from genomes selected from these two macrohaplogroups (Figure 2) showed a very similar expansion pattern, corroborating this hypothesis.

## Discussion

Our results strongly suggest that the demographic expansion putatively associated with the geographical expansion out of Central Asia and the initial peopling of Beringia as well as the estimation of ∼20 ky of occupation of Beringia by human groups before they entered the Americas [20] is merely a database artifact caused by the incorporation of mtDNA genomes of non-Native American ancestry in the analysis. Because the mtDNA haplogroups and sub-haplogroups have a strong and extensively studied geographic association e.g., [5], it is possible to identify almost unambiguously the ancestry of most haplotypes [15]. While this may be also true for the Y-chromosome [25], it is certainly not for most other nuclear markers, which typically display low levels of interpopulation differentiation and extensive haplotype sharing among populations e.g., [26], [27]. Possible applications of the BSP to autosomal or sex-linked haplotypes must carefully select the sampled populations to avoid incorporating into the analysis those recently introduced by admixture. Our analyses suggest that even a relatively small proportion of 12% (9/77) of “admixed” (or misassigned) haplotypes may significantly bias the overall result.

The population expansion that began 60–55 kya when non-Native American haplotypes are incorporated into the analysis most likely reflects, at least in part, the early expansion and diversification of macrohaplogroups M and N in Eurasia [28], which is unrelated to the specific process of the peopling of Beringia. As a consequence, Kitchen et al.'s estimation of a period of ∼20 ky of population occupation in Beringia based on the time interval between the “two expansions” is meaningless in the context of the peopling of Beringia or the Americas. In addition, it is important to stress that, because the mtDNA haplogroups currently in America represent derivations of both macrohaplogroup M (C, D) and N (A, B, X) e.g., [14], their TMRCA reflects the TMRCA of macrohaplogroups M and N in Asia (∼60 kya) [28]. Therefore, the >40 ky of constant population size found in the corrected data sets extending from the LGM bottleneck to the past to the TMRCA of all Native American mtDNA haplogroups does not offer any detailed view of the demographic history of Native Americans before the bottleneck. The genetic bottleneck associated with human isolation in Beringia [29], [30] may have erased from the recent non-Beringian Native American mtDNA data most of the details of its pre-Beringian demographic history. In this regard, discerning the population size changes during this period would mostly require acquiring mtDNA information of ancient samples from this time.

Interestingly, an almost identical pattern of population size change was found with the Kitchen et al. corrected data set and our previous analyses of mostly Native South American mtDNAs [13]. These results, therefore, strongly corroborate the mtDNA scenario for the peopling of the Americas presented in Fagundes et al. [13] and the integrated model that we suggested elsewhere [31]. This model suggests that the ancestral population colonized Beringia more than five thousand years before the LGM, remained isolated there during LGM, and likely experienced a population reduction and loss of genetic diversity by drift. The strong population expansion shown to have started around the end of LGM (∼18 kya) probably reflects the fast migration south of the Laurentide and Cordilleran ice sheets. Taking into account that the ice-free corridor between the ice sheets had not opened completely by this time interval, and that it could not have supported a viable human population earlier than 14 kya [32], [33], these findings support a coastal route as the major pathway for the peopling of the Americas, in agreement with recent published data from a panel of STR markers [34] and archeological data [35], [36].

However, time estimates are dependent on the evolutionary rate used in the analysis. The mtDNA evolutionary rate that we used [17] has been the most extensively used estimate in studies of human evolution e.g., [13], [14], [28], [37]. Nevertheless, other calibrations are available, although they are usually faster than ours [16], [21], [24]. The use of an internal calibration [21] results in a rate similar to that used by Kitchen et al. [20], which pinpointed the post-LGM population growth at ∼16 kya. Another rate, which uses only synonymous substitutions [16], suggests a mean coalescent age of ∼14 ky for the major Native American haplogroups [12]. However, the corroboration of the human occupation of southern Chile ∼14.5 kya [36] strongly suggests that haplogroup coalescences and the expansion out of Beringia should have occurred >16 kya, implying that the faster rates are unlikely to be accurate. In any case, the choice of a rate affects only the absolute numeric estimates, and does not change the shape of the BSP. Thus, our refutation of the Kitchen et al. [20] model for the peopling of Beringia is independent of all the controversies about the correct mtDNA substitution rate e.g., [38]–[40].
